# Assessing the capacity of primary health care facilities in Nigeria to deliver eye health promotion: Results of a mixed-methods feasibility study

**DOI:** 10.1371/journal.pgph.0000645

**Published:** 2022-11-11

**Authors:** Ada Aghaji, Helen E. D. Burchett, Shaffa Hameed, Clare Gilbert

**Affiliations:** 1 Department of Ophthalmology, College of Medicine, University of Nigeria, Enugu, Nigeria; 2 Department of Public Health, Environments and Society, Faculty of Public Health and Policy, London School of Hygiene & Tropical Medicine, London, United Kingdom; 3 International Centre for Eye Health, London School of Hygiene & Tropical Medicine, London, United Kingdom; Instituto Nacional de Geriatria, MEXICO

## Abstract

Over 25 million people in sub-Saharan Africa are blind or visually impaired, the majority from avoidable causes. Health promotion and disease prevention are important strategies for eye health, through good governance, health literacy and increasing access to eye care services. To increase equity in access for eyecare services, the World Health Organization Africa Region developed a package of interventions for primary eye care, which includes health promotion. The aim of this study was to assess the capacity of the primary healthcare system to deliver health promotion for eye care in Nigeria. Mixed methods were used during a survey of 48 government-owned primary health care facilities in Anambra state, Nigeria: interviews with district health supervisors, facility staff and village health workers, and a desk review of policy documents for primary health care and eye care in Nigeria. Findings were benchmarked against the capacities needed to deliver health promotion agreed through a Delphi exercise and were analysed using the World Health Organization’s health system building blocks. Eye health promotion policies exist but are fragmented across different national health policies. Health promotion activities focussed on “mobilising” community members to access care provided in facilities, particularly for women of childbearing age and young children, and health education was limited. Only one in ten facilities engaged the elderly and a fifth delivered health promotion for eye care. Health promotion activities were supervised in 43.2% of facilities and transport to remote areas was limited. A robust eye health promotion strategy needs to be included in the National Eye Health Policy. The scope of existing health promotion will need to expand to include eye conditions and different age groups. Increasing eye health literacy should be emphasized. Governance, training health workers in eye health promotion, educational materials, and transport to visit communities will also be needed.

## Introduction

It is estimated that in sub-Saharan Africa (SSA), 25 million adults have vision loss and a further 51.6 million have uncorrected presbyopia [1]. In addition, over 400,000 children in SSA are blind [2] and twice that number are visually impaired. Over 90% of the causes of vision loss are avoidable [3] and are amenable to health promotion or can be prevented. Primary health care (PHC) activities in facilities and communities are the vehicles through which health promotion and disease prevention are typically delivered. For example, many of the causes of blindness in children are preventable, through measles immunization, ocular prophylaxis of the new-born and vitamin A supplementation, with health education to promote exclusive breast feeding and a vitamin A-rich diet. Children with treatable causes such as cataract and glaucoma, need to be detected early and referred to specialist services [4, 5]. Health promotion also has a key role to play through policies which ensure that these services are accessible, and by health education to empower mothers to make the right choices about their child’s eye health. In adults, refractive error, cataract and glaucoma are the most common causes of distance visual impairment while presbyopia causes near visual impairment. Although these conditions may not be treated at PHC level, they can be detected, and individuals counselled and referred for eye care. Creating awareness about these conditions and where to seek appropriate management may encourage community members to seek treatment. Trachoma, which is endemic in rural, northern Nigeria, is the most common cause of preventable vision loss in adults and has effective strategies for control. These entail facial and environmental hygiene, mass drug administration of azithromycin, and surgery for the sight-threatening lid complications. Diabetes and diabetic retinopathy are emerging epidemics in SSA particularly in urban settings and will require significant health promotion for their control. Many of the interventions for the control of visual impairment are highly cost effective, but in SSA many people become or remain blind as they do not access services even when they are available. Commonly reported barriers are cost [6–9], cultural and social factors, lack of trust in the services [6], and lack of knowledge and awareness that many eye conditions can be prevented or treated and where treatment is available [6, 7, 9]. Health promotion activities in eye health are key to overcoming these barriers.

The fundamental role of health promotion is to “*enable people to increase control over*, *and to improve their own health”* [10], as stated in the Ottawa Charter for Health Promotion in 1986. Recently, the objectives of health promotion have been realigned with the United Nation’s Sustainable Development Goals, in response to globalisation and climate change [11]. Although strategies for health promotion have changed over time, the basic principle remains the same. In 2016, at the Shanghai Declaration on health promotion, the World Health Organization (WHO) identified three pillars for the delivery of health promotion; good governance, health literacy and healthy cities [11]. Good governance entails implementing clear policies, developing regulations and legislation to make healthy choices accessible and affordable to all, and creating sustainable systems for society. Health literacy entails empowering individuals to make the healthiest choices and decisions for themselves and their families by increasing their knowledge and social skills [12]. Healthy cities involves prioritising policies that create synergies between health and other city policies, supporting cities to promote equity and social inclusion of their diverse populations and re-orienting health and social services to make them more equitable [11]. The concepts articulated in the three pillars of health promotion apply directly to primary health care (PHC), and consequently can be applied to primary eye health care (PEC), the eye health component of PHC. Governance and policies (which increase access to services for eye care), and health literacy could address the high prevalence of visual impairment in SSA.

**Fig 1:**
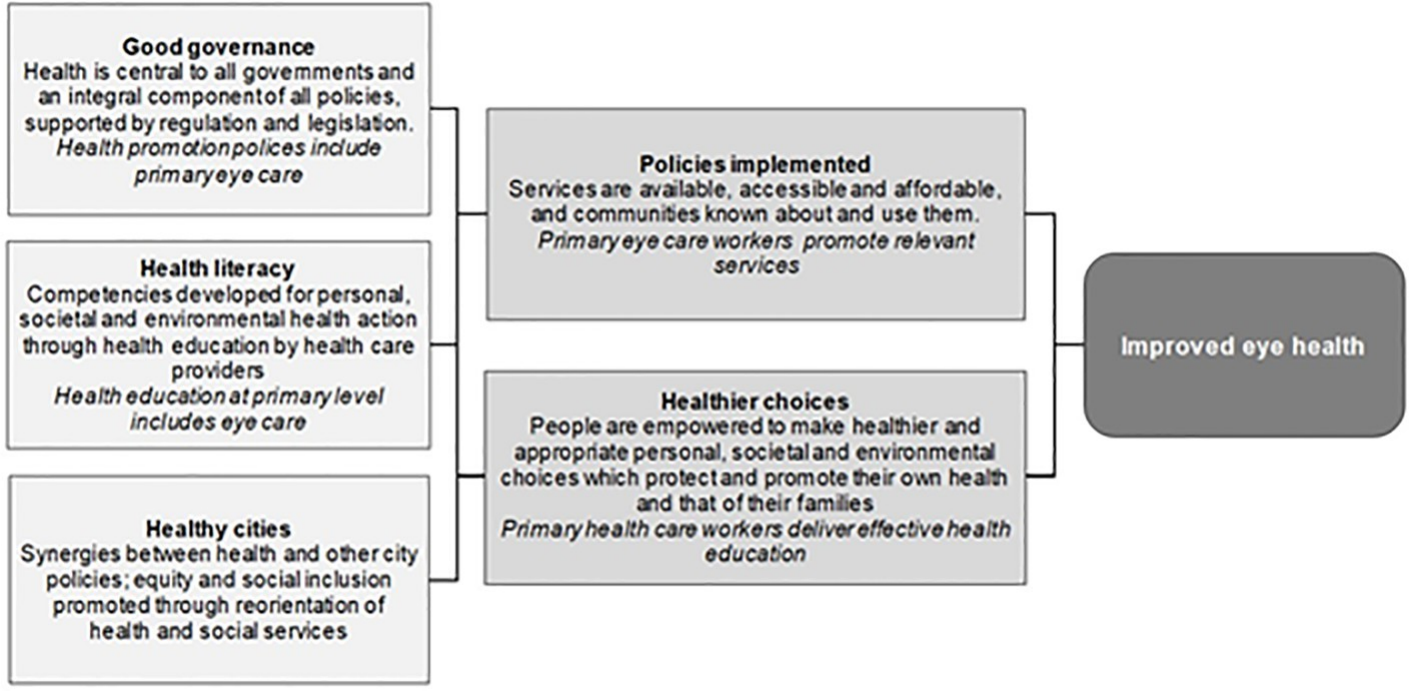
Conceptual model linking primary eye care to the World Health Organization’s three pillars of health promotion.

Concerning health literacy, PHC workers can promote relevant health services and in addition, provide appropriate health education messages to empower community members to make appropriate health choices and ultimately, have better eye health “Fig 1”.

In terms of healthy cities, re orienting health facilities to optimize access to health services and fostering social inclusion is crucial in reducing barriers to accessing healthcare [11].

Some of these health promotion strategies have been implemented in SSA. Data from 43 countries in SSA have shown that improving the quality of governance improves health outcomes in terms of reducing under five mortality rates, for example [13]. In SSA, mobile phone technology has been used as an intervention to improve health literacy for sexual reproductive health, maternal and child health, Ebola, tuberculosis and malaria [14]. In terms of healthy cities, health faciities located close to communities have shown higher odds of facility delivery for pregnant women and lower odds of neonatal mortality [15].

The WHO have developed a framework for the social determinants of health, which includes (i) governance and policies, (ii) social status and education, and (iii) material circumstances, i.e., living and working conditions [16]. The pillars of health promotion—governance, health literacy and healthy cities (where people live and work) bear a strong relationship with the social structures that determine equity and the health of populations. Effective eye health promotion could, therefore, play an important role in addressing inequity in eye health.

To increase access to eye care, the WHO Africa Region recently launched a package of interventions for PEC (WHO AFRO PEC package) which has two broad components: facility based management and eye health promotion [17]. WHO AFRO recommend that all aspects of their PEC package, including health promotion, be undertaken by suitably trained PHC staff. The health promotion component comprises specific eye health promotion messages, and the training curriculum includes how to give a health talk and how to counsel patients. The messages target mothers and caregivers of young children, people of all ages particularly the elderly, people with diabetes and relatives of adults with glaucoma. It is recommended that the health promotion messages be delivered using posters, health talks and in one-to-one counselling, and could include the use of mass media, such as radio, to reach communities [17]. However, before eye health promotion can be effectively delivered, it is important to determine the capacities needed to deliver health promotion, and the extent to which they are available in PHC facilities.

**Fig 2:**
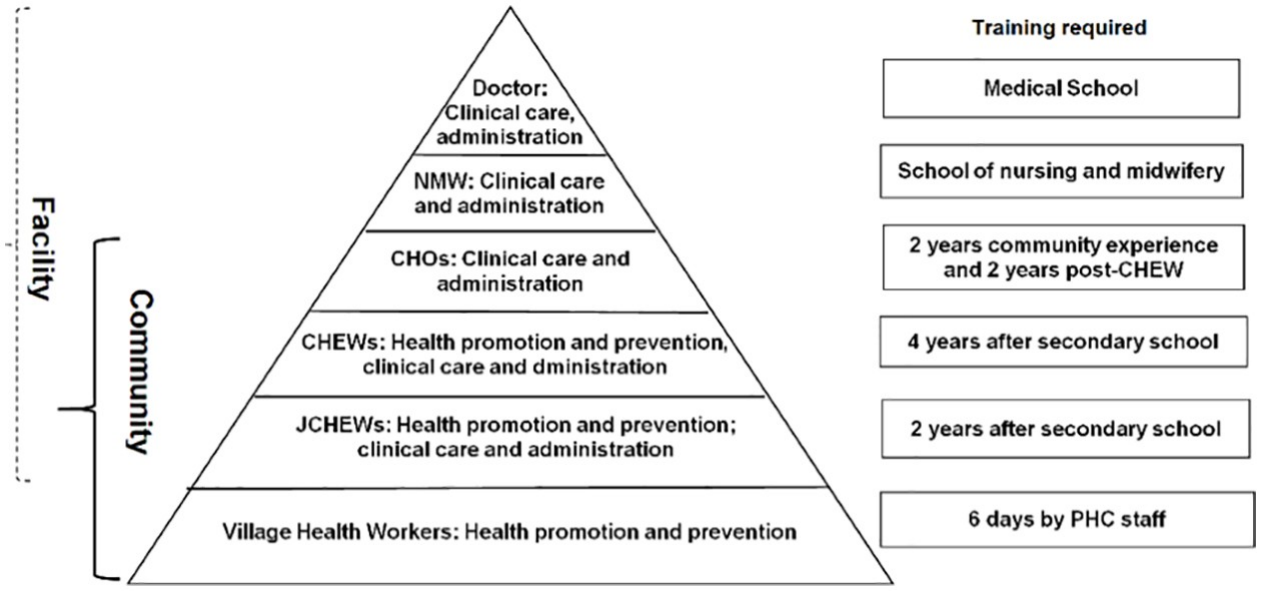
Roles of the different cadres of PHC staff [21].

In PHC facilities in Nigeria (health centres and health posts), health promotion is mainly delivered by junior community health extension workers (JCHEWs) and village health workers (VHWs). JCHEWs undergo two years of training in approved schools of health technology after completing secondary education. They are employed by the government, are attached to health centres or health posts, and spend 90% of their time in the community. VHWs are volunteers nominated and remunerated by the community; they are trained from six days to up to three weeks by the JCEHWs [18, 19]. Sometimes, health promotion may be conducted by community health extension workers (CHEWS) who undergo four years post-secondary school training in approved schools of health technology “Fig 2”. However, health promotion will require more than the human resource component to be effective. Key capacities required include relevant health promotion materials, supervision, transport, referral mechanisms, intersectoral communication and partnerships [20].

The overall purpose of this study was to assess capacity gaps in PHC facilities in Nigeria which would need to be addressed to effectively deliver the WHO AFRO PEC package. PHC capacity for facility case management has already been published [22, 23]. This paper reports the capacities available to deliver the health promotion component of the package.

## Methods

### Ethics statement

Ethical clearance was obtained from the National Research Ethics Committee of the Federal Ministry of Health, Nigeria, and the ethics review boards of the University of Nigeria Teaching Hospital and the London School of Hygiene & Tropical Medicine. Written informed consent was obtained from all participants and permission was taken to audio-record the interviews and to use anonymous quotes. All data collected were anonymised and every effort was made to ensure confidentiality.

### Overview

The study had several stages, which have been described in detail [21]. In brief, the methods included a literature review of PEC in SSA and of technical feasibility frameworks to identify a suitable framework to assess the feasibility of delivering PEC [24], In addition, a two-round Delphi exercise was conducted to provide consensus on the technical complexity of each component of the WHO AFRO PEC package using Gericke’s framework of technical complexity [24], and the capacities needed to implement each of them. These were then mapped onto the WHO health system building blocks. The capacities needed, which were derived from the agreed technical complexities, formed the basis of the data collection methods and tools, and guided the selection of relevant participant groups. We conducted a survey in PHC facilities, interviews with district PHC supervisors, facility staff and VHWs, and a desk review of policy documents for PHC and eye care in Nigeria to identify statements of relevance to health promotion for eye health. All the findings were mapped onto WHO’s health systems building blocks.

### Policy review

A desk review of national health policy documents that could support eye health promotion was undertaken. Relevant statements from specific policy documents were extracted and tabulated in a Microsoft Excel spreadsheet for analysis.

### Selection of PHC facilities and staff

The survey of PHC facilities (health centres and health posts) was conducted in Anambra state, Nigeria, between October 2018 and May 2019. Anambra is located in south-eastern Nigeria and has a population of 5.5 million [25]. The adult literacy rate is 78.2% compared with the national average of 62% [26]. There are 21 local government areas, or districts, which can be stratified into urban, semiurban, and rural. The main occupations are farming, manufacturing, and commerce as in many other states in Nigeria.

A representative sample of facilities was selected using two-stage stratified random sampling from a total of 235 PHC centres and 112 health posts. Six districts were selected to reflect their urban, rural or semi-urban location (ratio of 1:2:3, respectively). Within districts, facilities were selected to reflect the 2:1 ratio of health centres to health posts. Hence a representative sample of 33 health centres and 15 health posts was selected. Structured questionnaires and observational checklists were administered to one (J)CHEW in each facility by a trained research assistant. If a facility had more than one (J)CHEW, one was randomly selected. Nine facilities (three health posts and six health centres) were purposively selected based on an interim analysis to identify high and low performing facilities in relation to the health workforce, patient load and supervisory practices. In these facilities, facility heads (nurse-midwives, community health officers, or (J)CHEWs) were interviewed, and 18 VHWs (two VHWs attached to the selected facility) were administered a structured questionnaire to determine their health promotion practices. Finally, the supervisors for health in the six districts were interviewed using structured topic guides. Interview findings were triangulated with data from the observational checklists and structured questionnaires.

### Data management

Data from the questionnaires were entered into a custom-made database in Microsoft Access and transferred to STATA V.15.1 (Statcorp, Texas) using STATransfer for analysis. Frequency tables were generated from the data and simple descriptive analyses were performed. Interview recordings, which were all in English, were transcribed verbatim by AA and checked for accuracy. Analysis entailed re-reading the transcripts for familiarisation with the data which were then indexed, charted, mapped and interpreted by AA. Open Code Software V. 4.02 was used to assist analysis.

## Results

### Review of policy documents

The National Health Act (2014) and the National Health Policy (2016) support health promotion (Table 1). In addition, eye health promotion is specifically mentioned in the Nigeria Eye Health Strategic Plan (2014–2019), the National Eye Health Policy (2019) and the National Health Policy (2016). Furthermore, the National Strategic Health Development Plan II (2018–2022) specifies that an element of the eye health package at community and PHC level should include health education to promote eye health and disease prevention. In addition, the NSHDP 2018–2022 recommends promoting the establishment of a multi-sectoral coordination platform for eye health. However, eye health is not included in the most recent National Health Promotion Policy (2019).

**Table 1:** National health documents that support health promotion and eye health promotion in Nigeria.

Policy document	Policy for Health Promotion	Policy for eye health promotion
National Health Promotion Policy 2006 [27]	Health promotion priorities should reflect the health needs in Nigeria, including both communicable and non-communicable diseases like injury prevention, mental health and oral health Health promotion should empower individuals and communities to make informed decisions about their health	Not included
National Primary Health Care Development Agency; Guidelines for the development of PHC systems in Nigeria. 2012 [19]	The Minimum Health Package for Non-Communicable Diseases will include health promotion/education materials on non-communicable diseases displayed in all facilities. The health education programme will provide information on health promotion, disease prevention at community and individual levels. This requires creating awareness, demand and utilization/patronage of health services and programmes For the minimum package for health education and community mobilization, every facility should have relevant health promotion/ education materials conspicuously displayed in culturally acceptable language with graphics	Not included
National Health Act 2014 [28]	The National Council on Health, is the highest policy making body in Nigeria on matters relating to health, to promote health and healthy lifestyles	Not included
National Primary Health Care Development Agency Management Guideline For Primary Health Care Under One Roof 2016 [29]	The ward minimum health care package should include health promotion and community mobilisation	Not included
National Eye Health Policy 2016	Strengthen the capacity for health promotion at all levels	One of the activities is to improve public awareness of eye health
National Strategic Health Development Plan II. 2018–2022 [30]		An element of the eye health package at community and PHC level is health education for eye health promotion and disease prevention Strategic interventions to deliver eye care include: to strengthen advocacy, social mobilization and behaviour change communication on eye health and expand access (financial, geographical, social etc.) to comprehensive (promotive, preventive, curative and rehabilitative), appropriate and quality eye health services at all levels
Implementation guidelines for primary health care under one roof (PHCUOR) 2018 [31]	The Local Government Health Authority Management Team will include a programme officer for health promotion	Not included
Nigeria Eye Health Strategic Plan. 2014–2019 [32]		States to provide eye health education at community and PHC levels targeting those affected and those at risk of avoidable blindness. This should also focus on harmful eye practices, including *couching The National Eye Health Programme Secretariat will design and produce the eye health education materials
National Eye health Plan 2019		Healthcare facilities should deliver eye health promotion PHC workers should be trained to deliver eye health promotion Government will facilitate and regulate community participation in eye health promotion. Intersectoral collaboration will be done to improve eye health promotion

*Couching: a traditional method of cataract surgery that often results in poor visual outcomes.

### Facility survey

In the 48 facilities surveyed, all the heads of facility interviewed were female and representation was evenly distributed between urban, semi urban and rural facilities. However, four (J)CHEWs were not available; two were on study or maternity leave and two facilities did not have a (J)CHEW). All the (J)CHEWs were female, their mean age was 41.4 (SD± 8) years and 93.2% had completed training in schools of health technology.

**Fig 3:**
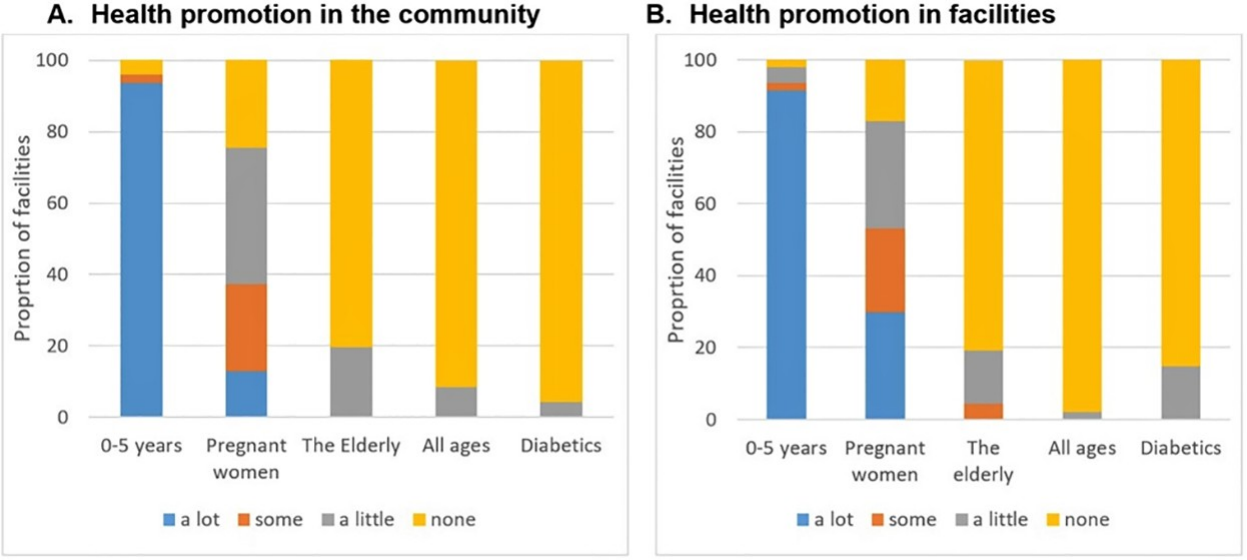
A and B amount of time staff spent with different groups on health promotion A in the community and B in facilities.

The majority of health promotion, whether delivered in communities or facilities, focussed on young children and pregnant women, with very little time spent on health promotion for the elderly, general health for people of all ages or for people with diabetes “Fig 3A and 3B”.

#### Health service data collection

There was no documented communication between the community and facilities as there were no referral slips, referral registers or evidence of two-way communication.

As there were some differences in health promotion activities undertaken in the community and in facilities they are described separately.

### Health promotion in the community

#### Human resources for health

Sixteen VHWs were interviewed as one facility had no VHW attached to it. Their mean age was 47.4 (SD±10) years and 25% were male. All the VHWs lived in the community, knew it very well and spoke the local language. The mean number of years they had lived in the community was 29.8 (SD±12) years.

Less than half of the 48 facilities had a VHW who conducted health promotion in the community, while over 80% of facilities reported that community health promotion was conducted by (J)CHEWs (Table 2). 25% of the VHWs interviewed said they rarely conducted health promotion activities.

**Table 2:** Health workforce for community health promotion.

	Health centre N = 32		Health post N = 12		Total N = 44	
Health workforce	N	%	N	%	N	%
Health promotion conducted by VHWs	14	43.8	5	41.7	19	43.2
Health promotion conducted by (J)CHEWs	28	87.5	9	75.0	37	84.1
(J)CHEW fluent in local language	31	96.9	12	100	43	97.7
(J)CHEWs knowledge of community: good/moderately good	27	84.4	12	100	39	88.6
(J)CHEWs confidence in delivering eye health promotion						
A little or more confident	14	43.8	7	58.3	21	47.7
Not confident	18	56.2	5	41.7	23	52.3
Willing to be trained on eye health promotion	30	93.8	12	100	42	95.5

Health promotion in the community should, ideally, be conducted by VHWs, but this was mainly undertaken by facility staff, including facility heads. The term frequently used by staff to describe health promotion was “mobilization”, by which they meant that they inform communities about the services available in the facility and encourage them to attend. The following comments from facility heads illustrate the work they do in communities:

*My main work is mobilisation*. *To mobilise the community…*.*because most of them don’t know what PHC is all about*. *So*, *my main work*, *my major work is to mobilise the community and tell them what PHC is all about*. *HoF/PHC/U/2*

*If there is a village meeting*, *we go there and inform them about the activities in the health centre and why they should come to the health centre… HoF/HP/R/7*

During the interview with one facility head it became clear that UNICEF had engaged their own VHWs to undertake health promotion on maternal and child health topics:

*[Concerning VHWs] the UNICEF group took two persons from each ward [community]…where they are mobilising people…*. *What they are mainly talking about to those people [community members] is family planning and immunisation*. *Those are the two major things they are mobilising people for*. *HoF/PHC/U/3*

It appears that a shortage of staff was hampering health promotion activities, as one head of facility explained:

*…Because we have a shortage of staff*, *we don’t do house to house mobilization*. *HoF/PHC/U/3*

Half (48%) of (J)CHEWs were confident about delivering eye health promotion and 96% were willing to undergo training (Table 2).

*Health promotion activities*. Health promotion activities by (J)CHEWS also mainly focused on mobilizing the community. For example, mothers of young children were informed that PHC facilities provide immunization and vitamin A supplements, or that medication was available for onchocerciasis, as explained by many heads of facility.

*I will tell them [the community] the services that are available like treatment of minor ailments*, *antenatal care*, *deliveries*, *immunisation services*, *giving drugs like Mectizan*, *Albendazole*, *treatment of onchocerciasis*, *treatment of childhood illnesses…… we mostly target women of child-bearing age and children under 5*, *though we do general mobilisation*. *HoF/PHC/U/2*

*We go there and inform them about important things like checking BP [blood pressure] and checking their sugar and other things*… *HoF/HP/R/7*

Most of the health promotion activities focussed on mothers and children (Table 3), which included health education about exclusive breast feeding. A key role of PHC staff was also to promote safe water and sanitation, and advise on household waste disposal, activities which were reported by a third to almost half of those interviewed.

**Table 3:** Health promotion activities in the community described by facility heads.

	Health centre N = 32		Health post N = 12		Total N = 44	
Frequency of health promotion by VHWs	N	%	N	%	N	%
At least weekly	4	12.5	3	25.0	7	15.9
At least monthly	3	9.4	3	25.0	6	13.6
Rarely/never	25	78.1	6	50.0	31	70.5
Community health promotion by (J)CHEWs:						
Measles immunisation	30	93.8	12	100	42	95.5
Vitamin A supplementation of young children	29	90.6	10	83.3	39	88.6
Exclusive infant breastfeeding	21	65.6	8	66.7	29	65.9
Diabetes prevention	8	25.0	2	16.7	10	22.7
Eye health	6	18.8	3	25.0	9	20.5
Care of the elderly	3	9.4	2	16.7	5	11.4
Safe water	12	37.5	3	25.0	15	34.1
Safe sanitation	15	46.9	5	41.7	20	45.5
Household waste disposal	16	50.0	2	16.7	18	40.9
How messages are communicated in the community						
Meetings with the target audience	29	90.6	12	100	41	93.2
Town criers/announcers	22	68.8	8	66.7	30	68.2
Home visits	8	25.0	3	25.0	11	25.0
Community leaders	7	21.9	1	8.3	8	18.2

A range of avenues were available for health workers to meet with target audiences in the community for health promotion, such as village meetings, and using town criers/announcers (Table 3), as two facility heads explained:

*We raised awareness by calling the announcer and telling him to tell the community that insecticide treated nets are available and they should come and get them here*. *HoF/HP/U/1*

*But I do go to their meetings*, *like the men’s meeting*, *the village men’s meeting*, *the village women’s meeting*, *I will attend*. *I talk to them*, *I tell them what*, *whatever I feel is conducive to them at that particular time*. *HoF/PHC/U/3*

#### Health promotion materials and transport

Over two thirds of facilities had health promotion materials for use in the community, but only a fifth were in the local language (Table 4). The commonest audiences for the posters were mothers of young children (63.6%) and pregnant women (54.5%) with none for people with diabetes or the elderly. The only education materials which mentioned eyes were posters on vitamin A supplementation.

**Table 4:** Materials for health promotion and target audiences.

	Health centre N = 32		Health post N = 12		Total N = 44	
	N	%	N	%	N	%
Ease of acquisition materials for all health promotion						
Fairly easy or easy	30	93.8	12	100	42	95.5
Difficult to acquire / not available	2	6.2	0	0	2	4.5
Materials for health promotion in the community						
Available	20	62.5	10	83.3	30	68.2
With explanatory graphics	19	59.4	10	83.3	29	65.9
In local languages	5	15.6	4	33.3	9	20.5
Available for the following target populations:						
Mothers with children 0–5 years	19	59.4	9	75.0	28	63.6
Pregnant women	13	40.6	9	75.0	24	54.5
All ages	3	9.4	3	25.0	6	13.6
People with diabetes	0	0	0	0	0	0
The elderly	0	0	0	0	0	0

Less than 10% of facilities had transport for PHC staff to visit communities. A head of facility commented on the challenges faced when visiting some hard-to-reach communities:

*We have gone across all the riverine and hard to reach areas*. *We went there and sensitised them…*‥*there is no finance for our transportation*. *We use a flying boat to cross over*. *HoF/PHC/R/9*

#### Governance

A higher proportion of health promotion activities conducted in the community by (J)CHEWs were supervised if they worked in health posts (25%) than in health centres (9.4%), and supervision of VHWs was only reported by two heads of health centres (6.3%). It became clear that (J)CHEWs were largely not aware of what the VHWs were doing, and they had limited control over their activities, as explained by two facility heads:

*No*, *nobody supervises them [VHWs]*. *The only way we know that they are doing it is that you may be here*, *and someone will come to me and say*, *“nurse somebody came to our meeting (in) our church and told us that this health centre now has a residential nurse*.*” That is the evidence that the VHWs are doing their work*. *HoF/PHC/U/2*

*[Concerning VHWs] They are invited*. *The government invites them*, *not me*. *They help in mobilising people in the communities to come out for immunisation*. *HoF/HP/U/1*

One of the reasons given for the lack of VHW supervision was that they no longer dispense medication:

*In those days we gave them [VHW] drugs…but now*, *because we don’t give them anything there is no more supervision*. *If you give them [drugs]*, *you supervise how they give the drug … but we encourage them to keep talking to people*. *HoF/PHC/U/3*

All the health promotion materials observed for use in the community had the official logos.

### Health promotion in facilities

#### Service delivery

Staff in all the facilities reported that they deliver health talks for measles immunisation and vitamin A supplementation but less than 10% of facilities gave health talks for the elderly or for the prevention of diabetes (Table 5). Only one facility, a health centre, had a list of topics to be covered in health talks, but this was not a weekly or monthly schedule.

**Table 5:** Materials to support health promotion in primary health care facilities.

	Health centre N = 32		Health post N = 12		Total N = 44	
**Leadership and Governance**	n	%	n	%	n	%
List of topics for health education in the facility	1	3.1	0	0	1	2.3
Supervision of health promotion in the facility	15	46.9	4	33.3	19	43.2
**Facilities that conduct health talks**	32	100	12	100	44	100
**Topics covered in health talks**						
Vitamin A supplementation of young children	32	100	12	100	44	100
Measles immunisation	32	100	12	100	44	100
Exclusive infant breastfeeding	30	93.8	6	50.0	36	81.8
Diabetes prevention	4	12.5	3	25.0	7	15.9
Care of the elderly	1	3.1	2	16.7	3	6.8
**Materials for health promotion in facilities**						
Available	32	100.0	12	100.0	44	100.
With explanatory graphics	30	93.8	11	91.7	41	93.2
In local languages	24	75.0	9	75.0	33	75.0
**Target populations for materials**						
Mothers of children 0–5 years*	28	87.5	12	100	40	90.9
Pregnant women	29	90.6	9	75.0	38	86.4
All ages	8	25.0	4	33.3	12	27.3
People with diabetes	0	0.0	0	0.0	0	0.0
The elderly	0	0.0	0	0.0	0	0.0

*Posters mentioned taking vitamin A for healthy eyes.

Staff also engaged in health promotion and health education talks in the facility, as explained by a facility head:

*But when they come to the health facility……we educate them*. *We give them a health talk on how to manage their lives and how to manage the present condition*. *HoF/PHC/U/2*

*What we do here……we give immunisation*, *health talks concerning prevention of malaria and to prevent common diseases*, *keep the environment clean to avoid mosquito bites*. *I tell them to use their nets to sleep*. HoF/HP/SU/6

Another facility head thought that delivering eye care in the facility would be likely to lead to a reduction in the use of traditional eye remedies:

*They use traditional medicines [for eye conditions]…*. *or if it is serious they go to [names two local towns]…*.*they squeeze them [local herbs] and drink them but if we start seeing eye patients here they will drop that practice and use orthodox medicine*. HoF/PHC/R/8

*Infrastructure*, *equipment and consumables*. Posters to support health promotion in facilities were similar to those for use in the community, but posters in facilities were more likely to be in the local language (75%), and a high proportion had illustrative graphics suitable for audiences with low levels of literacy. There were no health promotion posters for the elderly or for people with diabetes (Table 5).

*Governance*. A total of 19 JCHEWs (43.2%) reported that their health promotion activities in the facility were supervised, which was slightly higher in health centres (46.9%) than in health posts (33.3%) (Table 5).

## Discussion

### Policy gaps

Eye health promotion is key to delivering WHO’s Integrated People Centre Eye Care [33] and should be a crucial pillar of any eye health policy. However, our study shows that some although enabling national policies for eye health promotion are in place, these are limited, particularly at the PHC level. In addition, these policies are not being implemented possibly because they are fragmented across several other policies, and their relevance and importance may not be appreciated by non-eye care professionals. To address this, as recommended in the Nigeria National Strategic Health Development Plan II, 2018–2022 [30], as part of a multisectoral coordination platform for eye health, stakeholders from other health disciplines and non-health sectors need to work together to strengthen eye health promotion policies for the Nigeria National Eye Health Policy, addressing the current limitations particularly at the primary level. The resulting cohesive and comprehensive strategy would need to be ratified by the National Council on Health, the highest health policy making body in Nigeria, and relevant components integrated into other policies, such as child health, non-communicable diseases, care of the elderly and water and sanitation. The National Eye Health Policy is the main policy and therefore advocacy instrument for eye care. We recommend bringing together all eye health promotion strategies in the National Eye Health Policy because in South Africa, failure to implement eye health promotion at the primary level was attributed to the lack of a specific eye health promotion policy [34].

### Human resource development for eye health promotion

Appropriately trained health workers are crucial in the delivery of a health intervention, and workers skilled in health promotion should be available to deliver appropriate health education messages. However, imparting health education to the community, which would improve heath literacy, was limited in our study. Instead, the thrust of health promotion activities in the community was to increase uptake of the services provided in health facilities. The reason for the apparent lack of health education could be that staff lacked training in health education and behaviour change communication.

In our study, less than half of the (J)CHEWs were confident about delivering eye health promotion but almost all were willing to be trained in this area. Similar findings were reported in a study of eye health promotion in Ethiopia, where 47% of PHC workers were confident in delivering PEC and 75% felt that more training was needed [35]. Addressing this training capacity gap is crucial because health workers delivering health promotion need to be purposefully trained and have the necessary skills to deliver clear and relevant messages to the appropriate audiences [36, 37].

### Appropriate health promotion materials

Health promotion needs to be supported by appropriate educational materials. In our study, although health promotion materials were readily available and easy to acquire, over a third of facilities did not use them in the community and most were not in the local language. While this may have contributed to the lack of health education in communities, the majority of the posters had self-explanatory graphics. However, the focus of the health promotion posters was on maternal and child health. One of the posters, on vitamin A supplementation, included a message that supplements keep children’s eyes healthy, and there is scope for a similar message to be included in materials for measles immunization. Advocacy will be needed to ensure that messages for people with diabetes include that diabetes can causes serious eye complications, with messages which would increase their health literacy on what they could do to reduce the risk [38]. In addition, posters on eye conditions in older adults e.g. on presbyopia and cataract, could create awareness and stimulate conversations around accessing appropriate care for these conditions. More recently there have been calls for innovative methods of delivering health promotion messages [39]. A recent study in India demonstrated the effectiveness of using mobile phones to improve eye health literacy [40]. With increasing mobile phone penetration and internet connectivity in SSA, mobile phones could become an important tool for disseminating health promotion messages, including for eye health.

### Service delivery

To be effective, health promotion should be delivered to the appropriate target audience. Health promotion activities were mainly targeted at women of child-bearing age and their young children. Similar findings were reported in a study of community based health workers in Ethiopia where health promotion for measles immunisation, vitamin A supplementation and Crede’s prophylaxis was conducted by over 70% of PHC workers, while eye health promotion was conducted by less than a third [35]. Maternal and child health has been and remains a major and important focus of health promotion activities as a component of PHC. However, over the last decade or so there has been a global shift towards promoting the health of everyone in the community [41]. For health promotion to contribute towards a reduction in visual impairment and blindness, there needs to be an awareness of all the appropriate target groups. The elderly, who have the highest prevalence of blinding eye conditions, and people with diabetes who are at risk of diabetic retinopathy are very important target audiences. In addition, women, especially widows who have lower access to eye care in low- and middle-income countries (LMICs), should also be targeted [42]. Delivering targeted eye health promotion at community level may be one step on the ladder to reduce health inequities in accessing appropriate eye care. Indeed, health promotion has been identified as a key strategy to reduce health inequalities and it has been suggested that targeted health promotion should be prioritised [43]. In this study, the (J)CHEWs used a range of mechanisms to reach different target audiences, including talking to village, town criers and community leaders. One of the PHC workers in our study indicated that the topics of the health talks were spontaneous, depending on what seems to be relevant at the time. While this is a form of “people-centredness,” a prepared list of topics, including eye conditions, would ensure relevant health topics are covered. Other mechanisms for reaching the elderly and those with visual impairment could be informal providers of health care, such as Propriety Patent Medicine Vendors who are ubiquitous in Nigeria [44] and traditional healers, and religious leaders who played a key role in promoting the polio immunization campaign in Nigeria, for example [45]. One head of facility reported that oral herbal remedies were used to treat eye conditions in her community and suggested that introducing PEC may reduce their use. The use of traditional eye remedies is widespread in SSA, some of which are harmful [46]. In addition, the use of such remedies may delay seeking treatment from orthodox sources. This emphasizes the need for accessible eye care services and targeted eye health promotion.

### Supervision

Supervision is an important aspect of leadership and governance in health service delivery. However, supervision of health promotion in facilities and in the community was very limited. In the facilities, this may explain why none of the facilities had a schedule for health education talks and topics, with only one having a list of topics which should be covered in health talks.

Concerning community health promotion, VHWs were hardly supervised at all and the (J)CHEWs interviewed had very limited awareness of what the VHWs were doing. An explanation given for this was that VHWs do not require supervision as they are no longer permitted to treat patients. Lack of supervision may also explain why materials were not available in the local languages, particularly for activities in the community where it could be argued that they are more important. There was anecdotal evidence that a United Nations agency had appointed VHWs for health promotion, so by-passing the supervisory function of staff in health facilities. The fragmented structure of health promotion and its supervision may lead to the dissemination of disparate messages, and so there is need to develop a unified managerial structure for health promotion. Although there is conflicting evidence about the impact of supervision on health outcomes [47], it has been suggested that health system challenges, particularly a shortage of human resources in poor countries, make supportive supervision even more important, and that it is the quality of the supervision which is more important than the frequency [48].

### Infrastructure

Health promotion and its supervision in communities will require appropriate transport arrangements, which was a challenge in this study, particularly in hard-to-reach areas. Local transport will be needed by staff to visit communities [49] and lack of transport for field work in eye care has been a recurring problem [50]. Transport promotes access to health centres by community members as well as access to communities by health workers. Policy makers need to recognise the impact of lack of transport on the delivery of health services, and inter-sectoral collaborations which align health and transport policies, may help to address this. Creating synergies between health and transport policies aligns with the “healthy cities” policy of the Shanghai declaration on health promotion [11]. This is in line with the good governance pillar of the Shanghai Health Promotion Policy and the Nigerian health policy which encourage the promotion of inter-sectoral action for health and effective partnerships among all relevant stakeholders for health development by mainstreaming ‘Health-in-All’ policies [51].

### Generalizability to other settings

Eye health promotion, which encompasses good governance, health literacy and healthy cities with equitable access to health care, cannot exist in a vacuum and can only be embedded in existing health promotional structures. Although there are many critical gaps for health promotion, one of the strengths is the focus on maternal and childhood conditions, and eye health promotion for young children can be integrated into this. This can be an entry point for eye health promotion. Indeed, there are efforts underway to embed eye health and eye health prevention activities into WHO’s Integrated Management of Childhood Illnesses (IMCI) [52]. In many countries, eye health is not included in national health plans [33]. To implement and deliver eye health promotion effectively, eye health policies need to be intentional about including specific policies for PEC and eye health promotion which align with existing national policies. For SSA countries that will be implementing the WHO AFRO PEC, including the eye health promotion component, it is important to assess the capacity of the health system at the community and primary health care level to implement it.

### Strengths and limitations

To the best of our knowledge, this is the first study to assess the capacity of the health system to deliver eye health promotion. Assessment tools were developed based on a rigorous process that involved a review of the literature, appropriate technical capacity frameworks and consensus from experts in eye health in SSA using a Delphi exercise. The findings may be generalizable to other countries in SSA. A limitation of this study is that it did not assess community participation, which is central to the success of health promotion.

Further studies will be needed on the impact of health promotion on the uptake of eye care services in health facilities.

## Conclusions

Our study identified several capacity gaps in health promotion which will need to be addressed to implement eye health promotion effectively. A robust eye health promotion strategy needs to be included in the National Eye Health Policy. The scope of existing health promotion will need to expand to include those with eye conditions, the elderly and people with diabetes. This will require trained health workers, targeted health promotion supported by relevant health promotion materials in the local languages which are suitable for populations with low levels of literacy, transport to visit communities and supportive supervision.

## Supporting information

S1 Data JCHEW data form and checklist. (XLSX)

S2 Data JCHEW questionnaire. (XLSX)

S3 Data VHW questionnaire. (XLSX)

S4 Data Criteria for selection of facilities for semi structured interviews. (XLSX)

S1 Text Topic guide for facility heads and district level supervisors. (DOCX)

## Abbreviations

(J)CHEW: Junior Community Health Extension Worker / Community Health Extension Worker
HoF: Head of Facility
PEC: Primary Eye Care
PHC: Primary Health Care
SSA: Sub-Saharan Africa
VHW: Village Health Worker
WHO: World Health Organization
