# Supplementary material for: Assessing the capacity of primary health care facilities in Nigeria to deliver eye health promotion: Results of a mixed-methods feasibility study
Source: PLOS Glob Public Health. 2022 Nov 11;2(11):e0000645. doi: 10.1371/journal.pgph.0000645 (PMC10022001; doi:10.1371/journal.pgph.0000645)
Supplement: S1 Text — (DOCX) [file pgph.0000645.s005.docx]

**Topic Guide: District Level Supervisors**

**Aim: To assess (i) the extent to which eye health is supervised or can be supervised by district supervisors (ii) the perceptions/experiences of supervisors about PEC implementation in the district.**

How long have you worked here?

What is your main role in this district?

Next, I would like to ask you some questions about your role as a supervisor.

**Leadership and Governance**

- How do you carry out the supervision of primary health facilities in this district?
  - *Probe for how often each facility is supervised, how often (s)he goes on supervisory visits.*
  - *Are supervisory visits regular? Planned or impromptu?*
  - *Is there a supervisor’s manual for District level supervision? Probe for what activities are supervised.*
  - *Probe for what have been the challenges for supervision e.g. transport, time, funding. Ask for personal examples.*
  - *What do you usually do during a supervisory visit? (check register, drugs in store; teaching, observation of case management; solving problems; giving feedback and recommendations)*
- Do you supervise eye health activities at primary health care facilities in this district?
  - *If yes, what has been your experience?*
  - *If no, is there any reason why you don’t? Please explain.*
  - *What is your opinion about district level supervision for PEC in this district?*

***Human Resources for Health.***

- How would you describe your workload?
  - Probe for what happens on a normal working day.
- How confident are you about supervising PEC activities in this district?
  - *Probe: What training if any have you undergone in supervision of PEC?*
  - *Probe: What is your opinion of you and other district supervisors undergoing in-service training to supervise primary eye care in the districts?*
- Do most facilities have the full complement of staff?
  - *Probe: if not why not*
  - *Probe: it seems as if many facilities use volunteer CHEWs/JCHEWs. Why is this the case*
- What is staff turnover like in this district?
  - *Probe for how easy it is to recruit staff. Give an example of when this happened.*
  - *Is absenteeism a problem? Why?*
- How is in-service training of newly recruited staff done?
  - *Probe: Who does the training? How can staff to undergo in service training for eye care in this district?*
  - Probe: *What NGOs if any work in this community? How can they facilitate PEC training for health workers-VHWs, JCHEWs, CHEWs, etc?*

**Service Delivery**

- I understand that some districts in other states deliver PEC in their facilities. What do you think about making eye care available in community members at primary health facilities in this district? Why do you say so?
  - *Probe: What do you think will make it work?*
  - *What do you see as the problems to making PEC succeed?*
- I understand that community members sometimes seek eye care from inappropriate sources. What is your opinion on working with these sources to train them to refer patients to you? E.g. traditional health practitioners or patent medicine vendors.
- Is there any relationship between the health department and the education department at district level?
  - *Probe for how school eye health can be incorporated in schools?*

**Equipment, technology and consumables.**

- How are regular supplies of standard drugs and consumables ensured for facilities in this district?
  - *Probe: How regular or irregular is the supply?*
  - *Probe: What affects this supply?*
  - *Probe: How might eye drugs be supplied regularly to facilities in this district?*
- What processes would be required to supply equipment for eye care which is not currently listed as essential for PHCs, such as for examining the eyes or measuring vision?
  - *Probe for how basic eye equipment e.g. Snellen’s chart can be procured.*
  - *Probe for how new equipment is procured (sourced and supplied).*
- What processes would be required to supply medication for eye medication which not currently listed as essential for PHCs, such as for allergic eye disease?
- How are health promotion materials developed in this district?
- What processes wold be required to develop new health promotion materials for eye care?

**HMIS**

- What data do you collect from the primary health facilities in your district?
  - *Probe: Who determines what data is collected?*
  - *Who collects it?*
  - *What happens to this data*?
  - *How can eye care data be included in the data collected from the facilities?*

**Conclusion**

- What do you see as the main problems that might prevent primary eye care from being delivered well in this district?
- Name 3 of the most important things that would make delivery of primary eye care successful in your district?
- Finally, is there anything I have left out, or something you would like to mention regarding primary eye care in this district?

**Thank you for your time**

**Topic Guide: Head of Facility**

**Aim: To assess the perceptions/experiences of facility heads about PEC implementation in the facility.**

**Introduction**

How long have you worked here?

What is your main role in this facility?

Next, I would like to ask you some questions about your role as the Head of this Facility.

Please can you describe the activities which take place in this facility over the course of a usual week. For example, what happens on Mondays?

1. **Leadership and Governance**

- Do you supervise Health Promotion Activities in this community?
- If yes, what has your experience been like?
  - *Probe: What groups are targeted? e.g. new-borns, mothers and their young children, the elderly*
  - *Probe: What health promotion activities are supervised*
  - *Probe: What have been your challenges and opportunities. Ask for personal examples*
  - *Probe for how eye health activities are supervised*

1. **Human Resources for Eye Health**

- As a member of this community, can you tell me who people usually consult first when they have a health problem?
  - *What about people with eye conditions – who do they usually consult first? What do you think about that?*
  - *What would you feel about working with these groups to encourage them to refer eye patients to this facility?*
- Could you describe what happens when a patient with an eye problem comes to this facility? Give an example of when it happened.
  - *Probe for what knowledge and skills staff have to handle eye cases*
  - *Probe for how confident (s)he is in supervising primary eye care and what resources (s)he may need to effectively carry out primary eye care supervision-training, time, supervision*
  - *Probe for what in service training is available for health workers and whether any is available for primary eye care*

1. **Service Delivery**

- I understand that the facility sometimes produces key messages for health promotion. Can you explain the process of how you do this? Give examples from your personal experience
  - *What do you think about incorporating key messages for promoting eye health?*
  - *What has been your experience in demand creation for a new intervention? Give an example. e.g. insecticide treated mosquito nets*
- Are any NGOs supporting services in this facility or in the community?
  - *Probe: what do they support?*
  - *How effective has their support been?*
  - *In what way can these NGOs support eye care?*

1. **Equipment Technology Consumables.**

- Do you face any difficulties in sourcing equipment for this facility?
  - *Probe for how one gets equipment fixed when it breaks down*
- Based on your experience, do you think there will be any problems sourcing eye care equipment in this facility? *(Snellen distance visual acuity chart; near visual acuity chart, torches and batteries).*
- Do you have any problems with drugs being out of stock in this facility?
  - *Probe: how often are drugs out of stock?*
  - *Probe: what is done about this?*
  - *Probe for an example*
- How often are drugs you want to prescribe not provided in the central medical store?
  - *Probe: What do you do under these circumstances?*
- Based on your experience, are there problems sourcing consumables in this facility? *(saline, cotton wool, gauze, plaster)*
- How would easy will it be to stock medications for eye conditions in this facility?
  - *Probe for how easy it will be to stock eye medications, what the cost of medications are, what the demand for eye medications is like.*
- How is eye care medication dispensed to patients in this facility with eye conditions who need them?
  - *Probe for who prescribes the medication.*
  - *Probe for how much the drugs cost*
- Who in the facility is responsible for maintaining stocks of medication?
  - *Probe for what training if any they have had in this?*

1. **Health Management Information Systems**

I now would like to ask you some questions about the information you record in this facility.

- Please can you explain to me who is responsible for completing the patient registers?
- *Probe: How is patient attendance documented?*
- *Probe: Few eye conditions are registered in this facility (from Quant tool), why is this the case?*
- What is the process for referring patients to referral centres?
- *Probe for how compliant patients are.*
- *Probe for assisted referrals-phone calls or transportation.*
- *Probe in what way if any feedback is given from the referral centre.*
- In what way are staff shifts managed*?*
- *Probe: Is this a 24 hour facility?*
- Probe: Is there an inventory for drugs and consumables?
- *Probe: who is responsible for maintaining it*
- *Probe: whether staff have received training in inventory management.*
- I understand that you collate data on the number of patients who attend the facility to send to the district on a monthly basis. Does this include patients with eye conditions?
- *Probe for who determines what data are sent to the district supervisor.*

**Closing**

- How do you think demand for eye care in the community can be created?
- Some Primary Health Facilities in other states deliver primary eye care in their facilities. What is your opinion about that? Do you think it can work in your facility? Why do you say so?
- Name 3 of the most important things that would make delivery of primary eye care successful in your facility?
- Finally, is there anything I have left out or something else you would like to mention regarding primary eye care in this facility?
- If you think of something later, please feel free to contact me.

Thank you for your time
